# Supplementary material for: Phenotypic and Genetic Spectrum in 309 Consecutive Pediatric Patients with Inherited Retinal Disease
Source: Int J Mol Sci. 2024 Nov 14;25(22):12259. doi: 10.3390/ijms252212259 (PMC11595089; doi:10.3390/ijms252212259)
Supplement: Supplementary file 1 [file ijms-25-12259-s001.zip › ijms-3246842-supplementary.pdf]

Table S1. Genes implicated in the infantile group (0-6 years old at age of genetic diagnosis) but not in the juvenile cohort (7-17 years old at age of genetic diagnosis) as well as overlapping genotypes between the two age groups.

1. Genes associated with non syndromic IRDs

Genes unique to 0-6 year old, infantile cohort (11 genes)	Genes unique to the 7-17 year old, juvenile cohort (13 genes)
GPR143	ADAM9
OCA2	CNGA1
KCNV2	CREB1
LRP5	CTNNB1
NMNAT1	ELVOLF4
PDE6A	IMPG2
RPGRIP1	MFRP
SLC45A2	RD3
TYRP1	RP1
RPB3	RP1L1
CRB1	TRPM1
	TSPAN1
	VCAN

2. Genes associated with syndromic/systemic IRD

Genes unique to 0-6 year old, infantile cohort (19 genes)	Genes unique to the 7-17 year old, juvenile cohort (12 genes)
BBS1	BBS12
BBS2	CDH3
c5orf42	CEP120
CNNM4	CLN3
COL11A1	CLRN1
GLB1	COL18A1
HPS5	GRIN2A
LCHAD	HPSGNAT
LAMA1	KMT2D
LYST	WDR19
MMADHC	MT-TL1
NDP	KSS
PACS2	
PCDH15	
PMM2	
RNU4ATAC	
SCO1	
SCLT1	
TSC1	
TPP1	
